# Cyclin Y Is Involved in the Regulation of Adipogenesis and Lipid Production

**DOI:** 10.1371/journal.pone.0132721

**Published:** 2015-07-10

**Authors:** Weiwei An, Zhuzhen Zhang, Liyong Zeng, Ying Yang, Xueliang Zhu, Jiarui Wu

**Affiliations:** 1 Key Laboratory of Systems Biology, Institute of Biochemistry and Cell Biology, Shanghai Institutes for Biological Sciences, Chinese Academy of Sciences, Shanghai, 200031, China; 2 State Key Laboratory of Cell Biology, Institute of Biochemistry and Cell Biology, Shanghai Institutes for Biological Sciences, Chinese Academy of Sciences, Shanghai, 200031, China; 3 Shanghai Advanced Research Institute, Chinese Academy of Sciences, Shanghai, 201210, China; 4 School of Life Science and Technology, ShanghaiTech University, Shanghai, 201210, China; Institute for Nutritional Sciences, CHINA

## Abstract

A new member of the cyclin family cyclin Y (CCNY) is involved in the regulation of various physiological processes. In this study, the role of CCNY in energy metabolism was characterized. We found that compared with wild-type (WT) mice, *Ccny* knockout (KO) mice had both lower body weight and lower fat content. The *Ccny* KO mice also had a higher metabolic rate, resisted the stress of a high-fat diet, and were sensitive to calorie restriction. The expression levels of UCP1 and PGC1α were significantly higher in the brown adipose tissue (BAT) of the *Ccny* KO mice than that of the WT littermate controls, whereas there was no significant difference in BAT weight between the WT and the *Ccny* KO mice. In addition, the down-regulation of *Ccny* resulted in suppression of white adipocyte differentiation both *in vivo* and *in vitro*, while the expression of *Ccny* was up-regulated by C/EBPα. Furthermore, both hepatocytes and HepG2 cells that were depleted of *Ccny* were insensitive to insulin stimulation, consistent with the significant inhibition of insulin sensitivity in the liver of the *Ccny* KO mice, but no significant changes in WAT and muscle, indicating that CCNY is involved in regulating the hepatic insulin signaling pathway. The hepatic insulin resistance generated by *Ccny* depletion resulted in down-regulation of the sterol-regulatory element-binding protein (SREBP1) and fatty acid synthase (FASN). Together, these results provide a new link between CCNY and lipid metabolism in mice, and suggest that inhibition of CCNY may offer a therapeutic approach to obesity and diabetes.

## Introduction

Dysregulation of lipid metabolism results in many pathological disorders, such as type 2 diabetes, fatty liver, and cardiovascular disease [1–4]. Adipose tissue and the liver are the major effectors of lipid homeostasis, and they are mainly controlled by the insulin signaling pathway [5]. One of the important downstream targets that is regulated by the insulin pathway is the group of sterol regulatory element binding proteins (SREBPs). SREBPs belong to the basic helix-loop-helix leucine zipper (bHLH-LZ) family of transcription factors (TFs), which are capable of regulating the expression of many enzymes required for the hepatic biosynthesis of fatty acids, endogenous cholesterol and triglycerides [6,7]. There are three isoforms of SREBP transcription factors, SREBP1a, SREBP1c and SREBP-2. All three isoforms are synthesized as inactive precursors that are tethered to the endoplasmic reticulum membrane, and then cleaved to the mature forms when the sterol levels decrease or when there is insulin stimulation [8,9]. The mature forms of SREBPs translocate to the nucleus to activate the transcription of target genes [8]. Importantly, SREBP1c is expressed at particularly high levels in hepatocytes, and this expression is mainly regulated by insulin at the transcription level through AKT/PKB [10].

Cyclins are a family of cell cycle proteins that share a conserved region of approximately 100 amino acid residues, termed the cyclin box [11]. Cyclins bind specific CDKs through their cyclin box to form functional protein kinase complexes. Cyclin Y (CCNY) is a new member of the cyclin family that was originally cloned from a testis cDNA library [12] and was first characterized by its function in cell cycle regulation [13]. CCNY has been identified as a membrane-binding protein that can activate the kinase activity of cdk14 through direct binding to cdk14 [14]. In addition to its emblematic function of regulating the cell cycle, CCNY is also involved in many other cellular developmental processes. *Ccny* deletion impairs *Drosophila* development and mice spermatogenesis [15,16]. In the nervous system, *Ccny* drives synapse removal to regulate the maturation of neural circuits [17].

In the present study, by analyzing *Ccny* **-**deficient mice using a range of assays, we showed that CCNY is involved in adipogenesis and lipid accumulation in mice through regulation of the insulin signaling pathway and other regulatory pathways related to lipid homeostasis.

## Materials and Methods

### Animal studies

*Ccny* flox mice were generated by the Shanghai Research Center For Model Organisms (Shanghai, China). Crossing *Ccny* flox mice with EIIa-Cre mice generated *Ccny* heterozygous mice. The *Ccny* heterozygous mice were maintained on a mixed background. We backcrossed the mice for three generations with C57BL/6 mice purchased from SLAC laboratory (Shanghai, China). The *Ccny* KO mice were generated by crossing *Ccny* heterozygous mice. Tail biopsies of the mice were analyzed using genomic PCR. WT *Ccny* was detected using the primers P-F: 5′- AATACAGCTCTTGCTCCACCA-3′ and P-R: 5′- ATACAGCTCTTGCTCCACCA-3′. The PCR product was 400 bp in length. *Ccny* deletion was detected using primer P-F: 5’- GCTACCCGTGATATTGCTGAA-3’ and P-R: 5′- ATACAGCTCTTGCTCCACCA-3′. The PCR product was 700 bp in length.

The mice were fed either a normal chow diet or a high-fat diet (HFD) (Research Diets, Inc., NJ, USA) starting at 8 weeks of age. The HFD was maintained for up to 12 weeks. The body fat content was determined by nuclear magnetic resonance using a Minispec LF50 nuclear magnetic resonance analyzer (Bruker Optics). For calorie restriction, the mice were fed 90% of the normal food intake every day for a period of four weeks. At the end of the studies, serum and tissues were collected for further analysis. For *in vivo* studies of insulin signaling, male mice were fasted overnight, followed by an IP injection of insulin (5 U/kg body weight). The mice were humanely destroyed after 5 minutes, and the liver, white adipose tissue and muscle were excised and snap frozen for immunoblotting.

### Plasmids, antibodies, and reagents

The wild-type C/EBPβ, C/EBPα fragments were generated from the mouse cDNA library using the PCR method. The fragments were cloned into pcDNA3-HA vector.

We purchased the following antibodies: antibody against CCNY from Abcam (Cambridge, MA, USA); antibodies against phospho-insulin receptor β (Tyr1150/1151), AKT (11E7), phospho-AKT (Ser473), GSK3β, phospho-GSK3β (Ser9), UCP1 and PGC1α from Cell Signaling Technology (Beverly, MA, USA); antibodies against SREBP1 (K-10), PPARγ, C/EBPα, aP2 and the secondary antibody (horseradish peroxidase-conjugated anti-rabbit and anti-mouse IgG and donkey anti-goat IgG) from Santa Cruz Biotechnology (Santa Cruz, CA, USA); anti-FASN from R&D Systems (Minneapolis, MN, USA).

LabAssay Triglyceride and LabAssay Cholesterol kits were purchased from Wako (Japan). A free fatty acid quantification kit was purchased from Biovision (CA. USA). Mouse insulin ELISA kit was purchased from Mercodia (Uppsala, Sweden). Mouse neuropeptide-Y ELISA kit was purchased from Mlbio (Shanghai, China). Insulin, dexamethasone (DEX), 1-methyl-3-isobutylxanthine and oil red O were purchased from Sigma Aldrich (St. Louis, MO, USA).

### Cell culture and transfection

HepG2 cells (American Type Culture Collection, Manassas, VA, USA) and primary hepatocytes were cultured in 5.5 mmol glucose Dulbecco’s modified Eagle’s medium (DMEM) (GIBCO, Life Technologies, NY, USA) supplemented with 10% FBS and 1% penicillin/streptomycin. HEK293T (American Type Culture Collection) and 3T3-L1 cells (American Type Culture Collection) were kept in DMEM supplemented with 10% FBS and 1% penicillin/streptomycin. All cells were kept at 37°C in 5% CO_2_. Transient transfection was performed using lipofectamine reagent (Invitrogen, Life Technologies, NY, USA).

### Measurement of the metabolic rate and activity

The metabolic rate and locomotion were measured using a Comprehensive Laboratory Animal Monitoring System (CLAMS) (Columbus Instruments). After the mice acclimated to the system for 24 hours, the oxygen consumption (VO_2_), carbon dioxide production (VCO_2_), respiratory exchange ratio (RER) and food intake were individually monitored for the subsequent 24 hours. Energy expenditure was normalized to body weight. The activity of the mice was monitored using x-axis beam breaks.

### Glucose tolerance test and insulin tolerance test

The GTT was performed as previously described [18]. Briefly, for the GTT, the mice were fasted overnight and injected with glucose at 2 g/kg body weight. For the ITT, the mice were fasted 4 hours and injected with insulin at 0.75 U/kg body weight. The blood glucose concentrations were measured 15, 30, 60, and 120 minutes after the IP injection.

### Paraffin sectioning and hematoxylin and eosin (H&E) staining

The white adipose tissue (WAT) was cut from 8-week-old mice and fixed overnight using 4% polyoxymethylene at 4°C. The fixed tissue was washed for 2 hours to remove the polyoxymethylene. Then, the tissue was dehydrated using ethanol and xylene. The tissue was embedded in paraffin and cut into slices. The slides were treated with xylene to remove the paraffin and with ethanol for rehydration. H&E staining was performed according to the protocol of Beyotime Institute of Biotechnology (JiangSu, China).

### Isolation of various primary cells from mice

The primary adipocytes and stromal vascular cells were isolated from the WAT tissue of the 8-week-old mice. The WAT tissue was placed into pre-chilled PBS and washed three times to remove the blood. Then, the floating tissue was transferred to a dish and quickly cut into pieces. The tissue was then washed another two times and transferred to a new tube for digestion with 1 mg/ml collagenase and 4% BSA in PBS. The tube was incubated at 37°C for 30 min and centrifuged at 1,000 rpm for 10 min. The supernatant was discarded, and the pellets were re-suspended using erythrocyte lysis buffer (154 mM NH_4_Cl, 10 mM KHCO_3_, and 0.1 mM EDTA) for 1 min to eliminate red blood cells. The cell suspension was filtered using a 70-μm filter, and the cells were centrifuged at 1,000 rpm for 10 min. The cells were re-suspended using DMEM with 10% FBS, and the medium was changed 4 h after incubation. Primary hepatocytes were isolated by infusing the mouse liver with collagenase buffer, and cells were filtered through a 100-μm filter. The cells were seeded on collagen-coated 6-well plates at a density of 1x10^6^/well.

### Adipocyte differentiation and detection with oil red O staining

The 3T3-L1 pre-adipocytes and primary pre-adipocytes of the mice were kept at 37°C in 10% CO_2_ and were grown to confluence for two days (called D0). Then, the cells were cultured with 1 mg/ml insulin, 1 mM DEX and 0.5 mM 1-methyl-3-isobutylxanthine in DMEM supplemented with 10% FBS for two days. Next, the cells were treated with fresh medium containing 1 mg/ml insulin, and the medium was changed every two days until day 8. The differentiated adipocytes were stained with oil red O. We extracted the oil red O in triglyceride droplets using 100% isopropanol and quantified them by measuring the OD at 510 nm.

### Stable *Ccny* knockdown cell lines

To establish stable *Ccny* knockdown cell lines, a retrovirus system was introduced based on the manufacturer’s recommendations (Clontech). The targeted sequences of *Ccny* were cloned into the pSiren-RetroQ vector, and the targeted sequences of *Ccny* and shuffle are shown in S1 Table. Retroviruses were produced by cotransfecting 293T cells with recombinant pSiren-RetroQ plasmids and pCL10A1 helper plasmid. Then, we infected the proliferating HepG2 and 3T3-L1 cells using the 293T cell culture supernatants, which were supplemented with 8 μg/ml polybrene and passed through a 0.45-mm filter. After 12 hours, the cell lines were selected in medium supplemented with 3 μg/ml puromycin.

### RNA extraction and RT-qPCR

The total RNA of the samples was isolated using TRIzol Reagent (Life Technologies). cDNA was reverse transcribed using a ReverTra Ace qPCR RT Kit (TOYOBO, Osaka, Japan). Quantitative PCR analysis was performed using an ABI Prism 7500 detection system (Life Technologies). The reaction buffer was SYBR Green (TOYOBO). GAPDH was simultaneously detected as a control.

### Chromatin immunoprecipitation (ChIP) assay

The ChIP assay was performed as previously described by Pei et al [19]. The primer sequences used for detecting the precipitated DNA are shown in S1 Table.

### Statistical analysis

All results were analyzed using Microsoft Excel software. Student’s t-test was used to compare the significance between two groups and was judged at *, P< 0.05; **, P<0.01; ***, P<0.001.

### Ethics statement

All animals were maintained in strict accordance with the guidelines of the Institutional Animal Care and Use Committee of the Institute of Biochemistry and Cell Biology. All animals were kept on a 12-hour light/dark cycle and were given ad libitum access to food and water. All of the experimental procedures were approved by the Chinese Academy Science ethics commission (Permit Number: SIBCB-NAF-14-001-s304-007). The mice were anesthetized with sodium pentobarbital after an overnight fast. All efforts were made to minimize suffering.

## Results

### *Ccny*-deficient mice showed dysfunctional lipid metabolism

**Fig 1:**
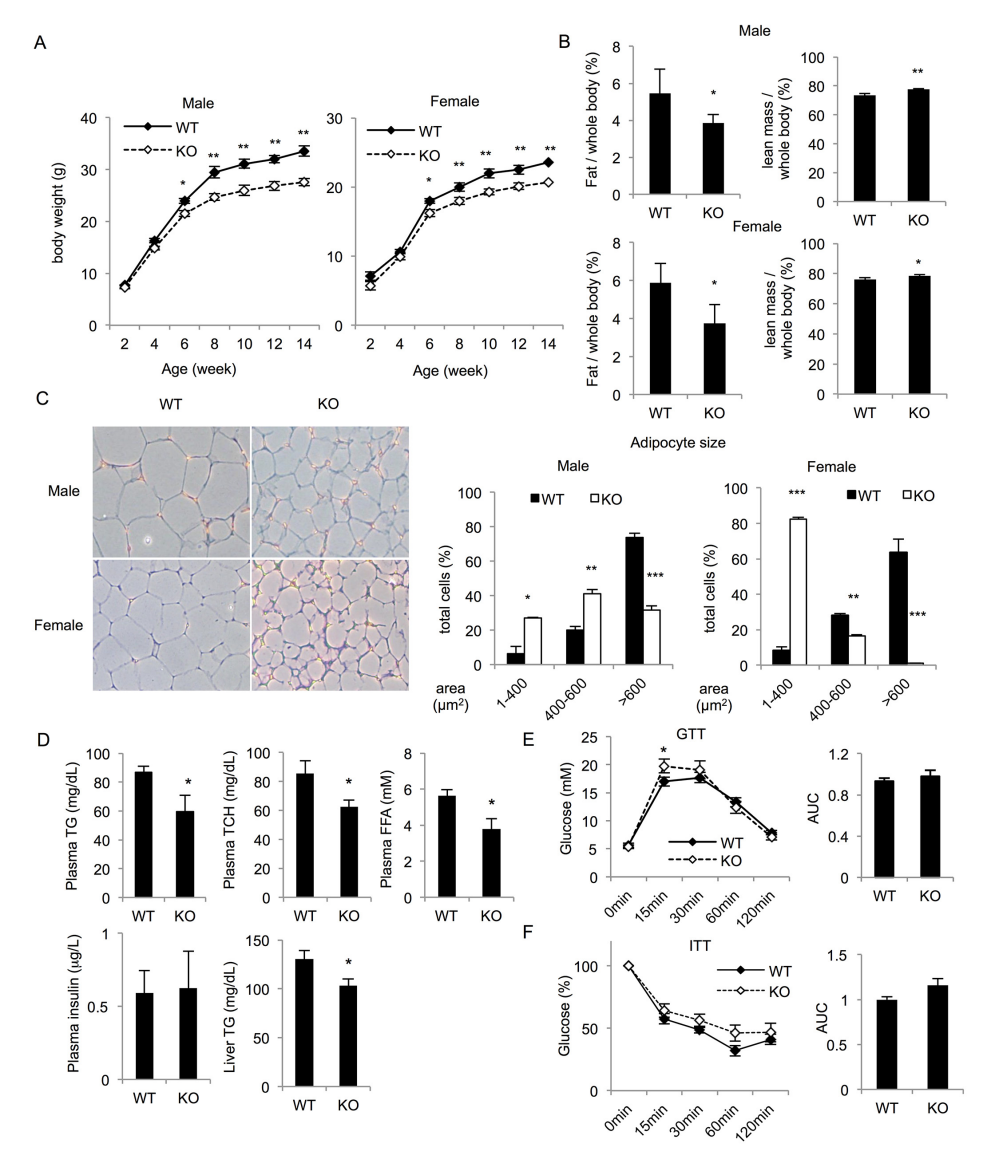
*Ccny* KO mice show dysfunctional lipid metabolism. The mice received a normal chow diet, and we measured parameters related to lipid homeostasis at the indicated times. (A) Growth curves of wild-type (WT) and *Ccny* KO mice (n = 7 male WT and n = 10 male *Ccny* KO; n = 7 female WT and n = 4 female *Ccny* KO). (B) Nuclear magnetic resonance analysis of the body fat and lean mass in 16-week-old *Ccny* KO mice and their littermate controls (n = 7 male WT and n = 10 male *Ccny* KO; n = 7 female WT and n = 4 female *Ccny* KO). (C) H&E staining of white adipose tissue in 16-week-old mice (left panel) and measurements of the adipocyte size (>500 cells per genotype) (right panel). (D) The levels of triglycerides, cholesterol, free fatty acids (FFAs), and insulin in fasting plasma and liver of the mice were measured in 16-week-old mice (n = 6 males per genotype). (E) Glucose tolerance test (GTT) (n = 6 males per genotype) (left), the area under the curve (AUC) (right). (F) Insulin tolerance test (ITT) (n = 7–8 males per genotype) (left), the area under the curve (AUC) (right). *, P<0.05; **, P<0.01; ***, P<0.001 WT vs *Ccny* KO. Error bars, S. E.

To investigate the potential role of CCNY in energy homeostasis, we first compared the body weight of the *Ccny* KO mice with the WT littermate controls during growth. The body weight did not significantly differ between the *Ccny* KO mice and the WT controls during the first 5 weeks; afterward, the *Ccny* KO mice weighed significantly less than the WT controls (Fig 1A). By 14 weeks, the *Ccny* KO males weighed a dramatic 20% less than the WT males (Fig 1A). Nuclear magnetic resonance analysis showed that the *Ccny* KO mice had a 30% decrease in total body fat mass compared with that of the WT controls; there was also a 6% increase in lean body mass (Fig 1B). In addition, the weights of the retroperitoneal, inguinal, perirenal and armpit fat pads in the *Ccny* KO mice were all lower than those in the WT controls (S1A and S1B Fig). Moreover, histologic analysis of the white adipose tissue (WAT) revealed that the *Ccny* KO mice had smaller adipocytes than those of the WT littermate controls (Fig 1C). Taken together, these results indicate that suppression of CCNY expression results in lipid metabolic dysfunction in mice.

Because WAT is an important endocrine organ and contributes to the metabolic state, a reduction in WAT might lead to lipodystrophy syndrome [4]. To assess the possibility of the lipodystrophic phenotype, we analyzed the total plasma- cholesterol, triglyceride, free fatty acid and liver-triglyceride levels. The plasma cholesterol, triglyceride, free fatty acid and liver-triglyceride levels decreased in the *Ccny* KO mice (Fig 1D). In contrast, the GTT assay showed no significant difference in the glucose tolerance between the *Ccny* KO mice and the WT controls (Fig 1E). And the ITT experiment also showed no significant difference in the insulin tolerance between the *Ccny* KO mice and the WT controls (Fig 1F). Additionally, there was no difference in the fasting basal glucose and insulin levels between the *Ccny* KO mice and the WT controls (Fig 1D and 1E).

**Fig 2:**
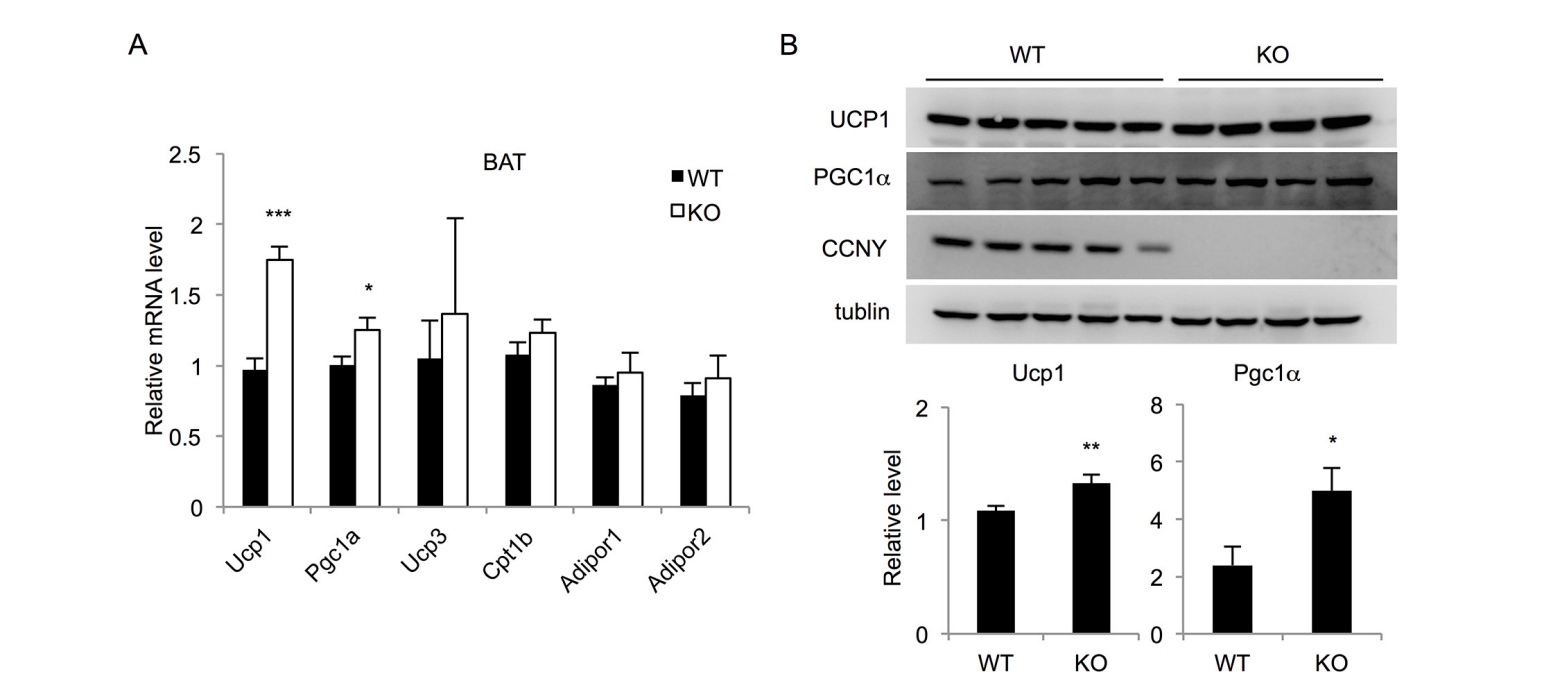
Increased expression of thermogenic genes in *Ccny* KO mice. (A) mRNA level of thermogenic genes in BAT and WAT (n = 4–5 females per genotype). (B) The protein level of UCP1 and PGC1α in BAT (n = 4–5 females per genotype). *, P<0.05; **, P<0.01; ***, P<0.001 WT vs *Ccny* KO. Error bars, S. E.

Using metabolic cages (Comprehensive Lab Animal Monitoring System), we analyzed the metabolic states of the mice. The VO_2_ (S2A Fig), VCO_2_ (S2B Fig), RER (S2C Fig), spontaneous locomotor activity (S2D Fig) increased significantly in the *Ccny* KO mice. These results suggest that the *Ccny* KO mice have increased energy expenditure compared with that of the WT controls. Therefore, we analyzed the weights of brown adipocyte tissue (BAT). The result showed there was no significant difference in BAT weights between the WT and *Ccny* KO mice (S1 Fig). On the other hand, both mRNA and protein levels of Ucp1, which is a mitochondria protein for energy dissipation, were significantly higher in the BAT of *Ccny* KO mice than that of their WT littermate controls (Fig 2). In addition, the expression level of transcriptional coactivator *Pgc1α*, a critical positive regulator of UCP1 expression, was also significantly upregulated in BAT of the *Ccny* KO mice (Fig 2). Taken together, these results suggest that the increased energy expenditure in the *Ccny* KO mice is at least partially due to a higher activity of brown adipocytes.

### *Ccny*-deficient mice are resistant to a HFD and sensitive to calorie restriction

**Fig 3:**
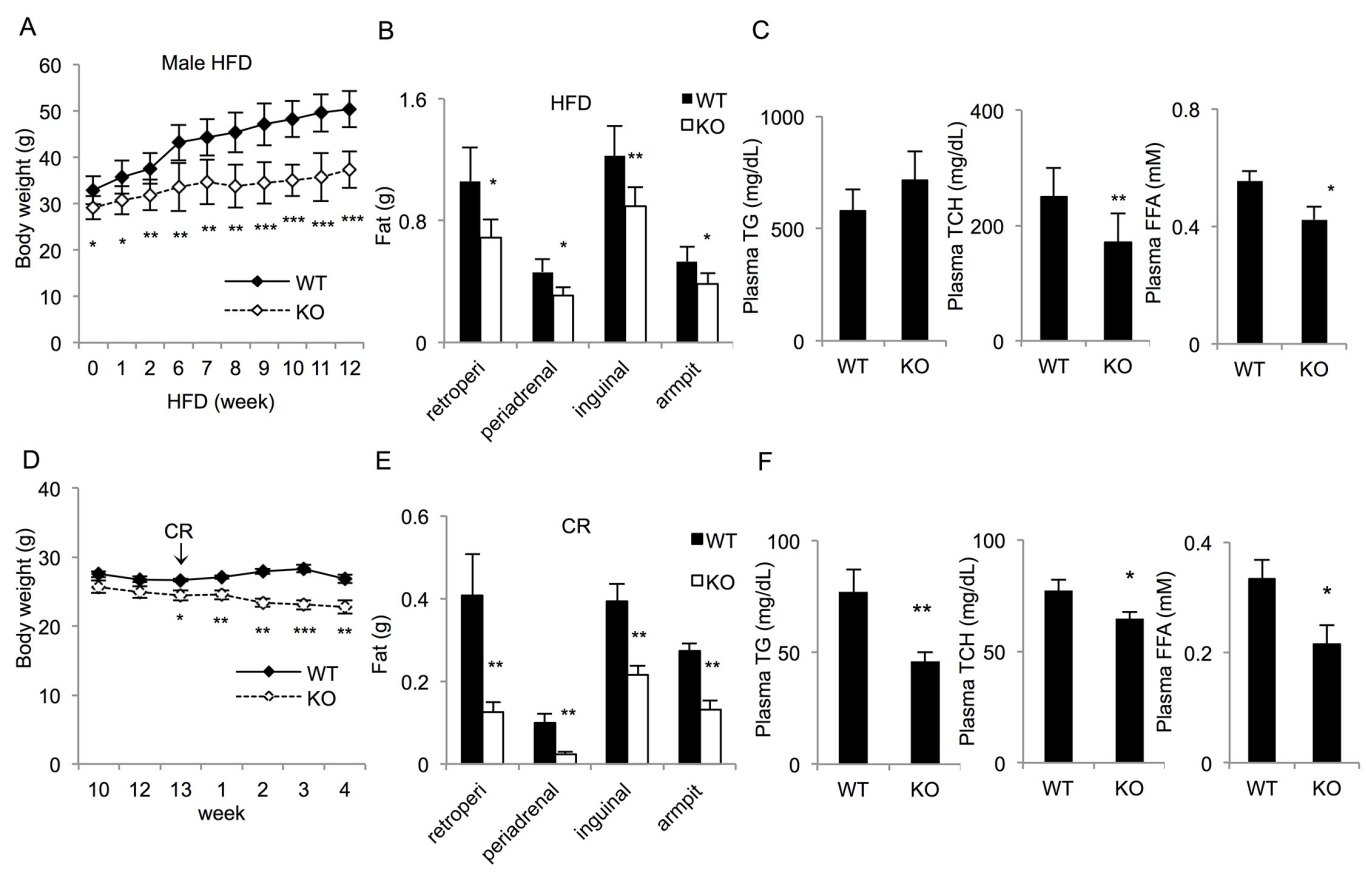
*Ccny* KO mice are resistant to a high-fat diet (HFD) and sensitive to calorie restriction. The mice received either HFD or calorie restriction. (A) The body weight curve of 8-week-old mice treated with 12 weeks HFD (n = 6–7 males per genotype). (B) Body fat contents of the 12-week-HFD mice (n = 6–7 males per genotype). Body fat content: retroperitoneal fat (retroperi), inguinal fat, periadrenal fat and armpit fat. (C) The fasting plasma levels of the triglycerides, cholesterol, free fatty acids (FFAs) were measured in 12-week-HFD mice (n = 6–7 males per genotype). (D-F) The mice were treated with calorie restriction. (D) The body weights of 13-week-old mice treated with a 10% reduced food intake were measured at the indicated times (n = 6–8 males per genotype); (E) Body fat contents (n = 6–8 males per genotype); (F) Fasting plasma levels of triglycerides, cholesterol, and free fatty acids (FFAs) (n = 6–8 males per genotype). *, P<0.05; **, P<0.01 WT vs *Ccny* KO. Error bars, S. E.

We expected that the *Ccny* KO mice and the WT controls would respond differently to energetic stresses. The mice were first subjected to a HFD. The body weight of the *Ccny* KO mice was lower than that of the WT control mice on the HFD (Fig 3A and S3A Fig). The body fat contents in the *Ccny* KO mice, including the retroperitoneal, inguinal, perirenal and armpit fat pads, were also smaller than that in the WT control mice under such treatments (Fig 3B and S3B Fig). In addition, the *Ccny* KO mice had greater variations in their triglyceride, total plasma cholesterol and free fatty acid levels compared with those of the WT controls (Fig 3C). These results suggest that the *Ccny* KO mice were resistant to the HFD treatment.

We also administered these two murine genotypes to calorie restriction. The 10% reduction in food intake could not lower the body weight of the WT mice (Fig 3D, also see ref. [20]), whereas the body weight of the *Ccny* KO mice decreased significantly under such treatments (Fig 3D). In addition, the calorie restriction also significantly decreased the body fat contents of the *Ccny* KO mice (Fig 3E). Furthermore, the *Ccny* KO mice had significantly lower triglyceride, total plasma cholesterol and free fatty acid levels compared with those of the WT controls on the calorie restriction (Fig 3F). Taken together, these results indicate that the *Ccny* KO mice are more sensitive to calorie restriction than the WT mice.

### CCNY is involved in adipocyte differentiation

**Fig 4:**
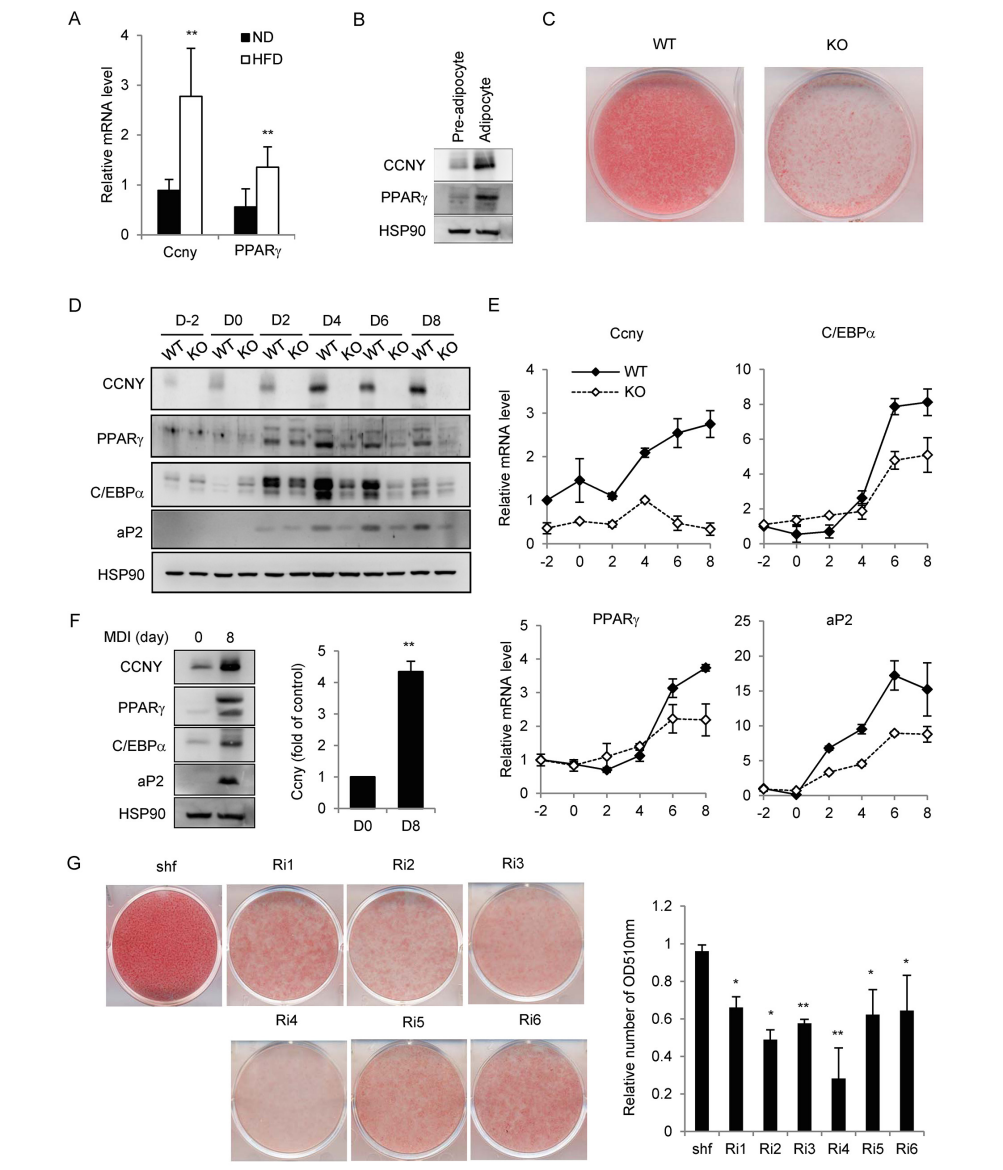
*Ccny* deficiency impairs adipogenesis *in vivo* and *in vitro*. (A) C57BL/6 mice (8 weeks old) were fed a HFD and normal diet (ND) for 13 weeks. The mRNA levels of *Ccny* and PPARγ in the white adipocyte tissue were detected. **, P<0.01 HFD vs ND. (B) Protein levels of CCNY and PPARγ in the primary pre-adipocytes and adipocytes of C57B6 mice. (C) The stromal vascular cells isolated from adipose tissue in *Ccny* KO and WT mice were differentiated into adipocytes. The cells were stained with oil red O on day 8. (D, E) The mRNA and protein levels of CCNY, PPARγ, C/EBPα, and aP2 were detected by real-time PCR and Western blot analysis, respectively. *, P<0.05. (F) 3T3-L1 cells before the MDI induction (D0) and 8 days after the MDI induction (D8). The protein levels of CCNY, PPARγ, C/EBPα and aP2 were detected by Western blot analysis (right) and quantified from three independent experiments (left). **, P<0.01 D8 vs D0. (G) 3T3-L1 cells subjected to siRNA treatments were stained with oil red O on day 8 (left) and quantified using measurements of the OD at 510 nm (right). The oil red O staining quantified results were normalized to control cells. *, P<0.05; **, P<0.01. The results shown here are representative of three independent experiments. Error bars, S. D.

We found that the *Ccny* mRNA level of the white adipocytes from the mice with HFD-induced obesity was significantly higher than that of the non-obese mice that consumed a normal diet; and the similar situation was also found for the adipogenesis-specific marker PPARγ (Fig 4A). In addition, comparing to preadipocytes, the CCNY protein level was elevated in the adipocytes that were differentiated from primary stromal vascular cells of the normal mice (Fig 4B). These results indicate that CCNY expression is positively associated with adipogenesis. Furthermore, the stromal vascular cells isolated from the adipose tissue of the *Ccny* KO mice also displayed impaired adipogenesis (Fig 4C), wherein the protein and mRNA levels of PPARγ, C/EBPα, and aP2 also declined (Fig 4D and 4E). Taken together, these results suggest that CCNY is required for the adipogenesis.

To further define the role of CCNY during adipogenesis, we used 3T3-L1 pre-adipocytes as an *in vitro* differentiation model [21]. The results showed that the CCNY expression in the induced adipocytes was much higher than that in pre-adipocytes, and other adipogenesis-specific markers, such as PPARγ, C/EBPα, and aP2 were also elevated in the adipocytes (Fig 4F). Next, we generated six stable *Ccny* knockdown 3T3-L1 cell lines through retrovirus-mediated shRNA targeting of six different regions of *Ccny* (see Materials and Methods). The *Ccny* knockdown efficiency was determined by the mRNA and protein levels of *Ccny*, with or without MDI induction (S4A and S4B Fig). These six stable cell lines and shuffle were then subjected to a standard adipogenic differentiation protocol. The oil red O staining assay showed that *Ccny* knockdown resulted in inhibition of the differentiation of 3T3-L1 pre-adipocytes (Fig 4G), suggesting that CCNY is needed for adipocyte differentiation.

Importantly, both the mRNA and protein levels of the middle and later adipogenesis-specific markers, including PPARγ, C/EBPα and aP2, were all inhibited in the *Ccny* knockdown cells (S4C and S4D Fig), whereas the early adipogenesis-specific markers, including C/EBPβ, C/EBPδ, KIf5 and Krox20, were not significantly changed in these cells (S4E Fig). These results suggest that CCNY does not function during the early stage of adipogenesis, although it is required for adipogenesis during the middle stage.

**Fig 5:**
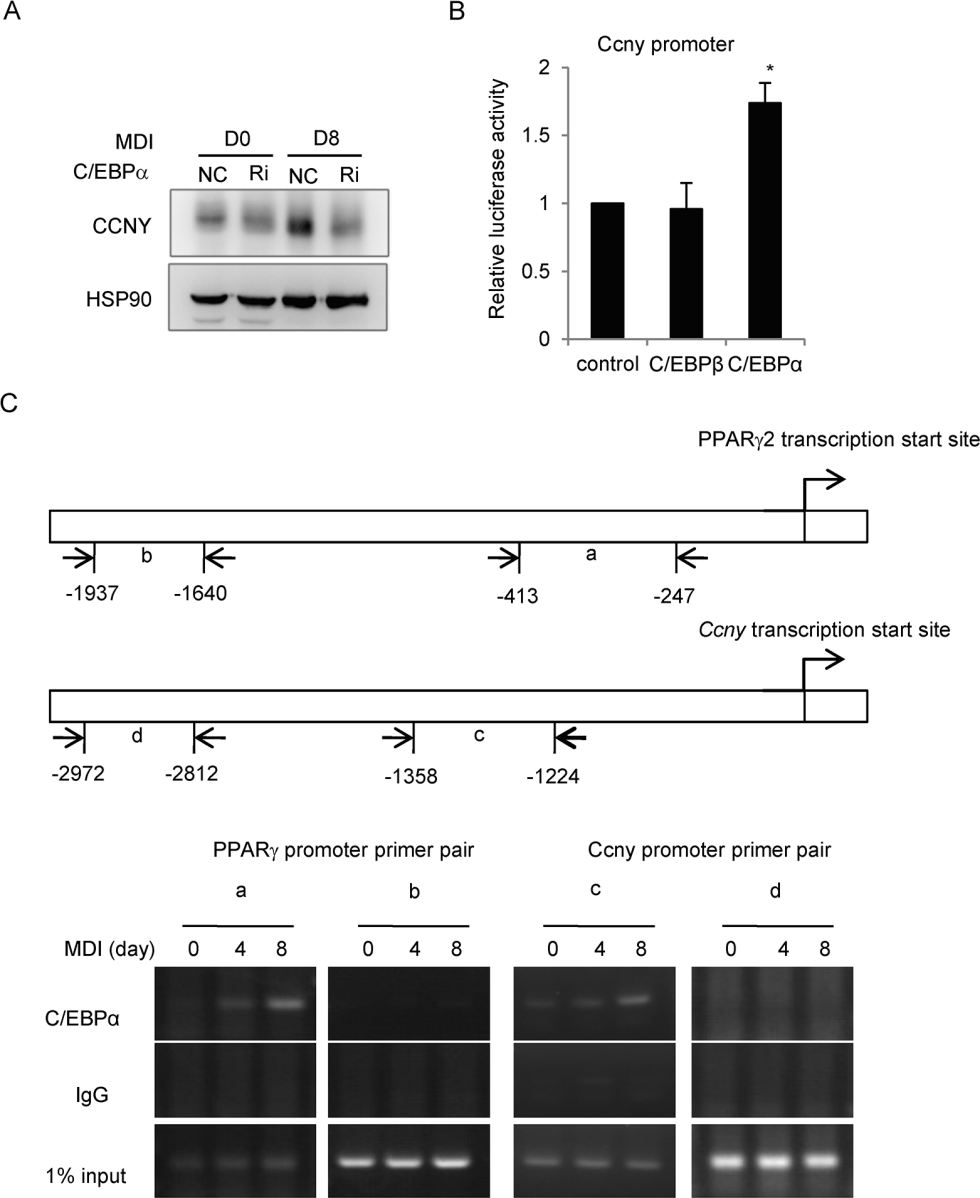
C/EBPα activates the transcription of *Ccny* during adipogenesis. (A) C/EBPα knockdown reduces the expression of *Ccny*. 3T3-L1 cells were stably transfected with C/EBPα-shRNA (Ri) and NC as control. The CCNY protein levels of the cells were detected on day 0 and day 8 after the MDI induction. Hsp90 is the loading control. The results shown here are representative of three independent experiments. (B) 293T cells were co-transfected with the pGL3-*Ccny* promoter and a C/EBPβ, C/EBPα or control vector. The results are expressed as the firefly luciferase activity and normalized to the Renilla luminescence. *, P<0.05 C/EBPα compared with control. Error bars, S. D. (C) C/EBPα binds to the *Ccny* promoter in 3T3-L1 cells. The MDI-induced 3T3-L1 cells at the indicated time points (days 0, 4 and 8) were subjected to ChIP with an anti-C/EBPα or IgG antibody. C/EBPα bound to the *Ccny* promoter (primer pair c) but not the primer pair d; the binding of the PPARγ2 promoter (primer pair a), but not the <-2,000 bp region (primer pair b), was detected as the control.

We further analyzed TFs that possibly interact with the promoter region of *Ccny* to regulate *Ccny* expression using a TF-prediction database (http://www.cbrc.jp/research/db/TFSEARCH.html), and found several potential TFs, including C/EBPα that is activated during the middle stage of adipogenesis [22]. Because *Ccny* was up-regulated during the middle stage of adipocyte differentiation (Fig 4D and 4E and S4C and S4D Fig), C/EBPα might be responsible for regulating *Ccny* expression. Therefore, we generated stable *C/ebp*α knockdown cells and found that the CCNY expression level in the *C/ebp*α knockdown cells was lower than that of the wild-type controls after the differentiation process (Fig 5A). In addition, a luciferase reporter assay showed that the *Ccny* transcription was elevated for the co-expression with C/EBPα but not C/EBPβ (Fig 5B). These results suggest that *Ccny* is a target of C/EBPα. Then, we performed chromatin immunoprecipitation (ChIP) assays to test whether C/EBPα could be directly bound to the endogenous *Ccny* promoter in 3T3-L1 cells during adipogenesis. The PPARγ promoter was detected at day 4 and peaked at day 8 after MDI induction; the *Ccny* promoter had similar binding activity (Fig 5C). However, these samples did not bind to the IgG antibody (Fig 5C). These results suggest that C/EBPα could directly bind to the *Ccny* promoter and activate the expression of *Ccny* during the middle stage of adipogenesis.

### CCNY is involved in insulin signaling and the regulation of its downstream targets

**Fig 6:**
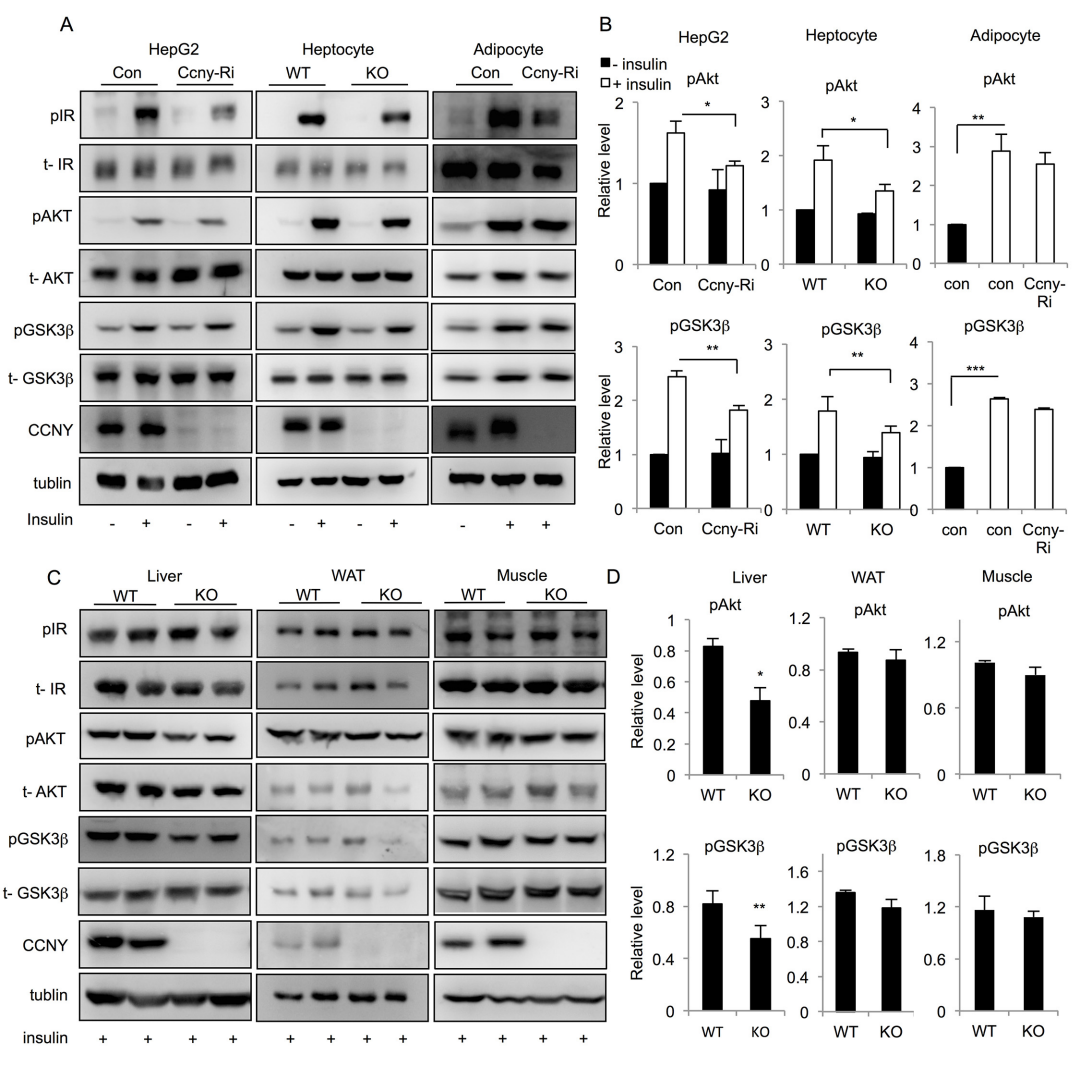
*Ccny* deficiency inhibits the activation of insulin signaling. Stable *Ccny* knockdown HepG2 cells, differentiated stable *Ccny* knockdown 3T3-L1 cells and primary hepatocytes of *Ccny* KO mice were stimulated with insulin for 15 min (HepG2, 100 nmol; 3T3-L1, 100 nmol; Hepatocytes, 10 nmol). (A) Western blot analysis of the phosphorylation of insulin receptor β (IRβ), AKT, and GSK3β. (B) The relative ratios of phosphorylated AKT and GSK3β were quantified (HepG2, from three independent experiments; 3T3-L1, from three independent experiments; Hepatocytes, n = 3). (C) *Ccny* KO mice and littermate controls (n = 2) of age 16 weeks were fasted overnight, IP injection of insulin (5U/kg body weight). The mice were humanely destroyed after 5 minutes and the liver, WAT and muscle were excised and used in western blotting analysis. (D) The relative ratios of phosphorylated AKT and GSK3β. *, P<0.05; **, P<0.01 WT vs *Ccny* KO, or con vs *Ccny*-Ri. Error bars, S. E.

The insulin signaling pathway plays an important role in regulating energy metabolism [23–25]. In the present study, we analyzed the insulin signaling pathway in stable *Ccny* knockdown HepG2 cells and differentiated 3T3-L1 cells using a retrovirus system and primary hepatocytes from *Ccny* KO mice. The results showed that the insulin-induced phosphorylation of AKT and GSK3β was partially inhibited in the *Ccny* knockdown HepG2 cells and primary hepatocytes from *Ccny* KO mice, but no such changes were detected in the 3T3-L1 adipocytes (Fig 6A and 6B), suggesting that CCNY is needed for the full function of the hepatic insulin signaling pathway. Furthermore, the insulin induced phosphorylation of AKT and GSK3β was decreased in the liver of the *Ccny* KO mice compared with that in the WT controls (Fig 6C and 6D). On the other hand, there were no significant differences in the insulin induced phosphorylation of AKT and GSK3β between the WAT and muscle of the *Ccny* KO mice and the WT controls (Fig 6D). Taken together, these results suggest that CCNY involves in the insulin signaling pathway mainly in mouse liver.

**Fig 7:**
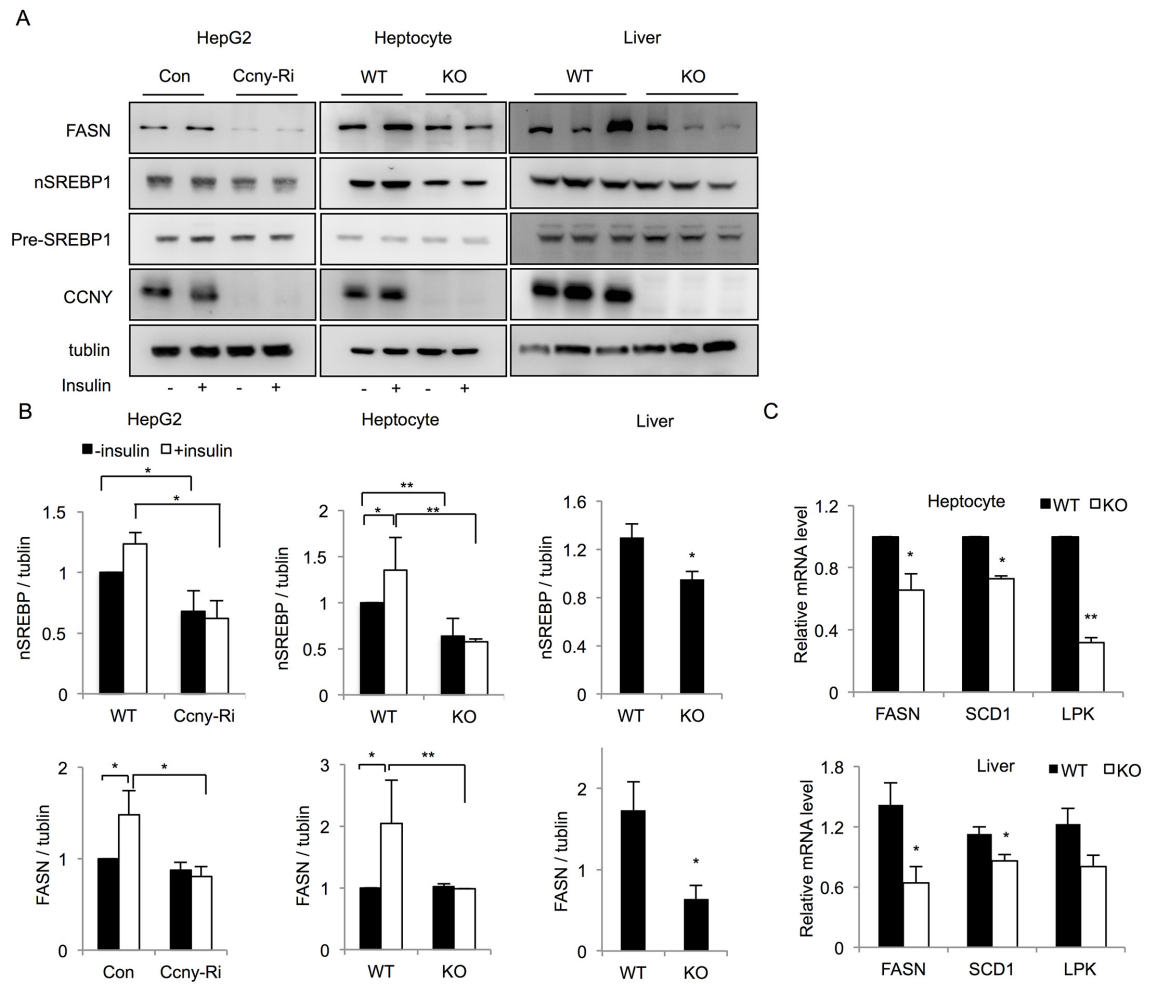
*Ccny* deficiency blocks the insulin-stimulated expression of target genes. (A) Western blot analysis of SREBP1 and FASN in stable *Ccny* knockdown (*Ccny*-Ri) HepG2 cells, hepatocytes and liver tissue from *Ccny* KO mice and WT controls. (B) The relative levels of SREBP1 and FASN were quantified (HepG2, from three independent experiments; Hepatocytes, n = 3; Liver tissue, n = 3). (C) Real-time PCR analysis of the mRNA levels of lipogenic genes in the hepatocytes and liver of WT and *Ccny* KO mice. *, P<0.05; **, P<0.01 WT vs *Ccny* KO, or con vs *Ccny*-Ri. Error bars, S. E.

Previous studies reported that insulin stimulation could activate the transcription of SREBP1 through the PI3K-AKT pathway [10]. We analyzed the protein level of SREBP1 in the stable *Ccny* knockdown HepG2 cells and the primary hepatocytes from the *Ccny* KO mice. The results showed that protein levels of the mature SREBP1 forms (nSREBP1) were lower in both *Ccny*-deficient cells, whereas there was no significant difference in the levels of the SREBP1 precursor (Pre-SREBP1) between the *Ccny*-deficient cells and the WT controls, with or without insulin stimulation (Fig 7A and 7B). Furthermore, we found that the insulin treatment significantly elevated the protein levels of nSREBP1 in the normal CCNY-expressing HepG2 cells or hepatocytes, whereas the insulin treatment did not increase the levels of nSREBP1 in both *Ccny*-deficient cells (Fig 7B). Taken together, these results suggest that CCNY promotes the insulin-induced maturation of SREBP1 mainly in the liver cells.

Because fatty acid synthase (FASN) is a downstream target of SREBP1, we also analyzed the FASN expression under the same conditions. The results showed that the protein level of FASN in the HepG2 cells and the WT hepatocytes was significantly higher than that in both *Ccny*-knockdown HepG2 cells and the hepatocytes of *Ccny* KO mice (Fig 7A and 7B). We further analyzed the expression of FASN and SREBP in the liver of the *Ccny* KO mice, and got the similar results (Fig 7A and 7B). In addition, we analyzed the mRNA levels of *Fasn*, stearoyl-CoA desaturase-1 (*Scd-1*) and L-pyruvate kinase (*Lpk*), which are downstream targets of SREBP1. The mRNA levels of these three enzymes in the *Ccny* KO hepatocytes and liver tissue were all significantly lower than that in the WT hepatocytes and liver (Fig 7C). Taken together, these results suggest that CCNY is required for the activation of the insulin signaling pathway, functional insulin signaling results in the elevation of mature SREBP1, and SREBP1 up-regulates the expression of downstream genes such as FASN and SCD-1.

## Discussion

### CCNY involves in regulation of energy expenditure

Based on analyzing the metabolic states of the mice using metabolic cages, we showed that the *Ccny* KO mice had a significant increase in food intake compared to that of the WT controls (S2E Fig). So we detected the level of neuropeptide-Y (NPY), which acts as the neurotransmitter involving the regulation of the food intake [26]. The result showed that the level of NPY in the *Ccny* KO mice was a little bit higher than that in the WT controls, while this difference did not show statistical significance (S2F Fig).

Previous studies showed that NPY and other hormones involved in the regulation of activity or development of brown adipocytes [27–29], particularly, the knockdown of NPY expression promoted the development of brown adipocytes [27]. So we also analyzed BAT development of the mice, and found there was similar BAT weight between the WT and *Ccny* KO mice (S1 Fig), which is consistent with the result there was no significant difference of NPY level between the *Ccny* KO mice and the WT controls (S2F Fig). On the other hand, our results showed that the expression levels of UCP1 and its transcriptional coactivator *Pgc1α* were significantly higher in BAT of the *Ccny* KO mice than that of the WT littermate controls (Fig 2). It is proposed that multi-factors from gut and brain participate in the regulation of the BAT activity [30], and the present data suggest that CCNY might also involve in regulating the BAT activity.

Since previous reports showed that the BAT thermogenesis is tightly correlated with the food intake in rodents [31,32], we propose that the up-regulation of BAT activity in the *Ccny* KO mice elevates energy expenditure and partially results in the increase of the food intake (S2E Fig). Taken together, increase of physical activity (S2D Fig) and BAT activity in the *Ccny* KO mice could generate the hyperphagic behaviors.

### CCNY promotes differentiation of white adipocytes

The present study shows that the mRNA and protein levels of CCNY are up-regulated in adipocytes, both *in vivo* and *in vitro* (Fig 4A, 4B and 4F), and this increase might be induced by C/EBPα during the middle stage of 3T3-L1 pre-adipocyte differentiation (Fig 5). Moreover, the *Ccny*-deficient cells have a reduced capacity to differentiate into adipocytes, both *in vivo* and *in vitro* (Fig 4C and 4G). Therefore, these results indicate that CCNY plays a positive role in adipogenesis.

Because a number of cell cycle proteins are devoted to adipogenesis through regulation of clonal expansion during adipogenesis [33,34], we analyzed the CCNY effects on 3T3-L1 cell proliferation. There was no significant difference in the proliferation rate between the *Ccny* knockdown cells and the control cells at the exponential growth and clonal expansion stages (S5 Fig), indicating a cell-cycle independent role for CCNY during adipogenesis.

Since previous studies showed that CCNY was mainly located in the cell membrane [14–17], we suspect that CCNY regulates adipogenesis through its interaction with other adipogenesis-related signaling pathways. A previous report showed that CCNY in the membrane could activate Wnt signaling through phosphorylation of the co-receptor LRP6 [35]. Importantly, Wnt signaling is involved in the regulation of adipogenesis [36,37]. Therefore, future studies should pay attention to the relationship between CCNY and cell signaling pathways that regulate adipocyte differentiation.

### CCNY mainly involves in regulating insulin signaling pathway of mouse liver

Insulin signaling controls metabolism of glucose and lipid in peripheral tissues of animals, particularly in the liver, white adipose tissue (WAT) and skeletal muscle. Importantly, insulin signaling regulates various components in those different tissues, e. g. insulin facilitates GLUT4 translocation from intracellular sites to the plasma membrane of fat or muscle cells, but promotes GLUT2 translocation in the liver cells [38]. The present study showed that insulin signaling pathway of both the *Ccny*-deficient HepG2 cells and the primary hepatocytes derived from the *Ccny* KO mice was impaired in response to the insulin stimulation, whereas no such defect of insulin signaling was detected in the *Ccny*-knockdown adipocytes (Fig 6A and 6B). The data that liver but not WAT and muscle from the *Ccny* KO mice presented insulin resistance (Fig 6C and 6D) further support this observation at the cell-level, suggesting that CCNY is mainly required for the functional insulin signaling in mouse liver. The future studies should focus on the molecular mechanism of CCNY regulating the function of insulin signaling in mouse liver, which might help us to understand how CCNY selectively functions in insulin signaling pathway of different peripheral tissues.

It is known that insulin signaling pathway controls the activity of a lipid regulator SREBP1 [8,39,40]. SREBP1 is regulated at three levels, the transcriptional level, the proteolytic cleavage level and the post-translational modification level [9, 41]. The present results indicate that CCNY in the hepatic cells or tissue regulates the mature forms of SREBP1 after proteolytic cleavage instead of regulating the expression level of pre-SREBP1 (Fig 7A and 7B). Because the administration of insulin only resulted in a significant increase in mature SREBP1 in CCNY-positive cells but not CCNY-deficient cells (Fig 7B), we propose that CCNY regulates the maturation of SREBP1 through activation of the insulin signaling pathway. This idea is supported by our present observations that CCNY deficiency resulted in partial inhibition of AKT and GSK3β (Fig 6A and 6B). However, the relationship between CCNY and insulin signaling requires further investigation. It is worth analyzing whether CCNY can co-localize with the insulin receptor and directly bind to members of the insulin signaling pathway. Although insulin signaling was impaired in liver of the *Ccny* KO mice (Fig 6), the GTT and ITT showed no significant changes between the *Ccny* KO mice and the WT controls (Fig 1E and 1F). In addition, there was no difference in the fasting glucose and insulin levels between the *Ccny* KO mice and the WT controls (Fig 1D and 1E). Two identified features in the present study should be considered for this phenotype of normal glucose metabolism in the *Ccny* KO mice. Firstly, the CCNY-deficiency generated the impeded adipogenesis of WAT (Fig 1B and 1C; Fig 4), which might result in lean body mass (Fig 1B; S1 Fig) and resistant to the HFD treatment (Fig 3A and 3B; S4A and S4B Fig). Secondly, the CCNY-deficiency resulted in the up-regulation of BAT activity (Fig 2), which could elevate energy expenditure in the *Ccny* KO mice (S2 Fig), in addition to the increase of physical activity (S2D Fig).

In conclusion, the present studies indicate that CCNY might play diversified roles in the different peripheral tissues of mice, and the interactions among these CCNY-deficient peripheral tissues might compensate each other for regulating the glucose and lipid metabolism in the body.

## Supporting Information

S1 Fig The body fat content of *Ccny* KO mice is lower than that of WT mice. (TIF)

S2 Fig Elevated energy expenditure in *Ccny* KO mice. (TIF)

S3 Fig Female *Ccny* KO mice are resistant to high-fat diet (HFD)-induced obesity. (TIF)

S4 Fig *Ccny* knockdown deregulates the expression of adipogenesis marker proteins. (TIF)

S5 Fig *Ccny* knockdown has no influence on the cell cycle. (TIF)

S1 Table The primer pairs used in the text. (DOCX)
