# Supplementary figures and images for: Cyclin Y Is Involved in the Regulation of Adipogenesis and Lipid Production
Source: PLoS One. 2015 Jul 10;10(7):e0132721. doi: 10.1371/journal.pone.0132721 (PMC4498623; doi:10.1371/journal.pone.0132721)

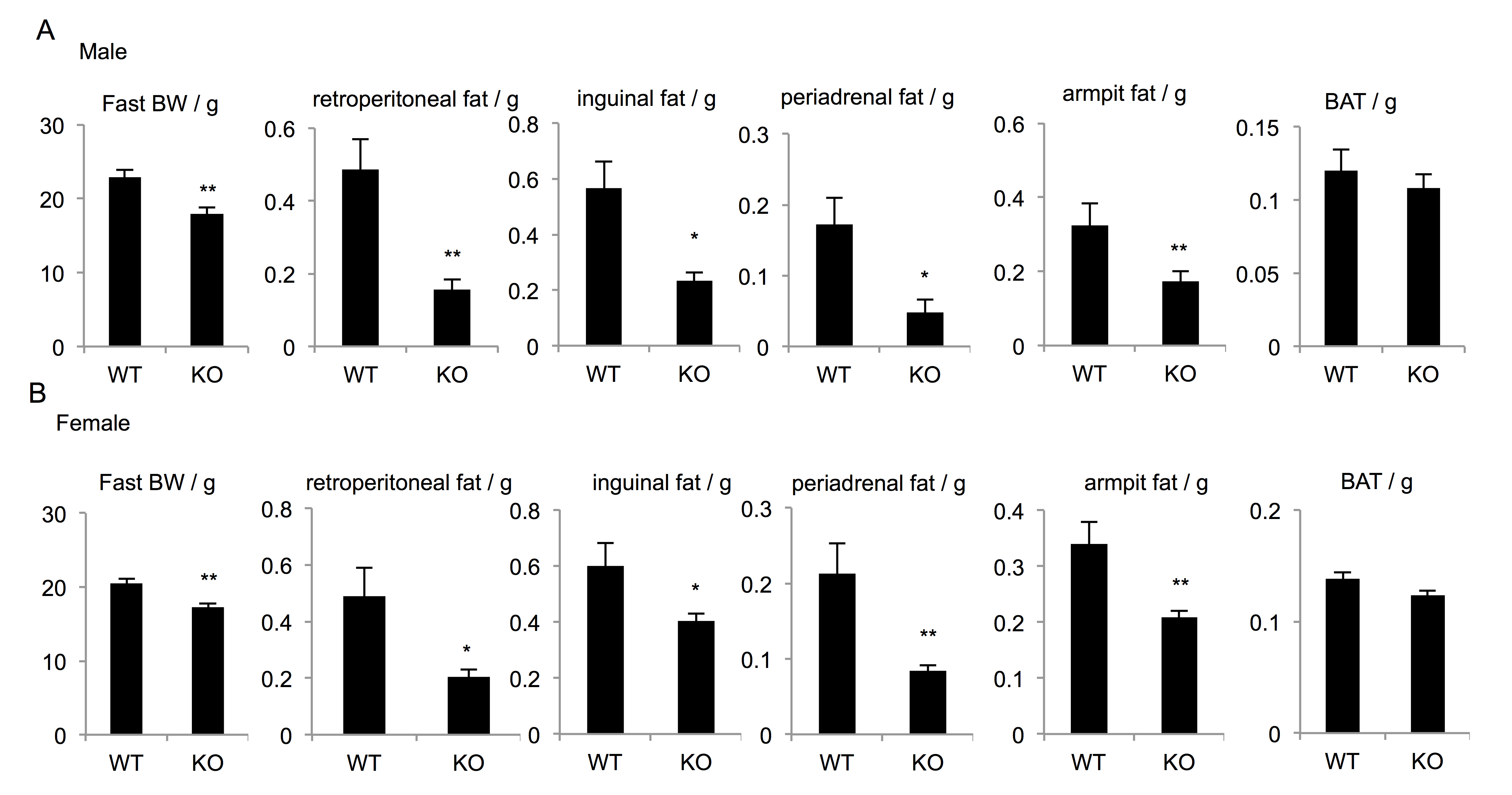

Supplement: S1 Fig — (TIF) [file pone.0132721.s001.tif]

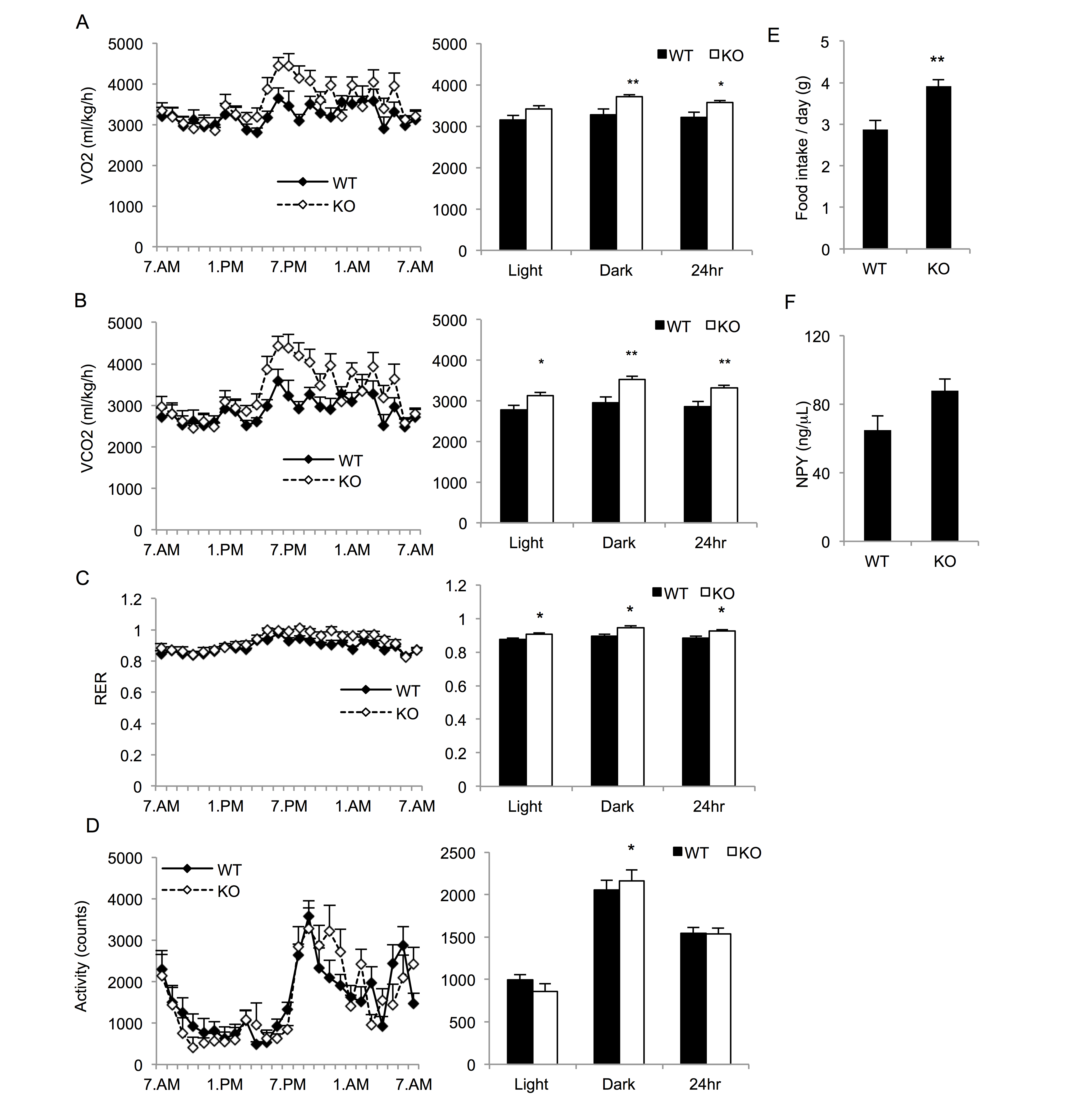

Supplement: S2 Fig — (TIF) [file pone.0132721.s002.tif]

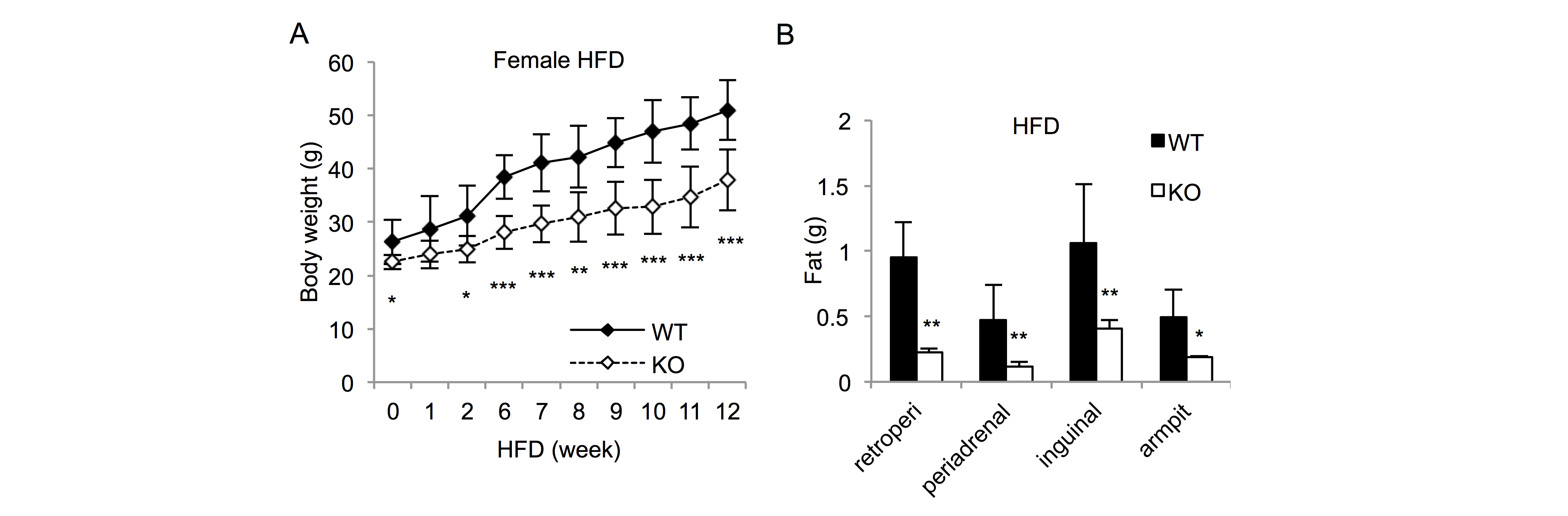

Supplement: S3 Fig — (TIF) [file pone.0132721.s003.tif]

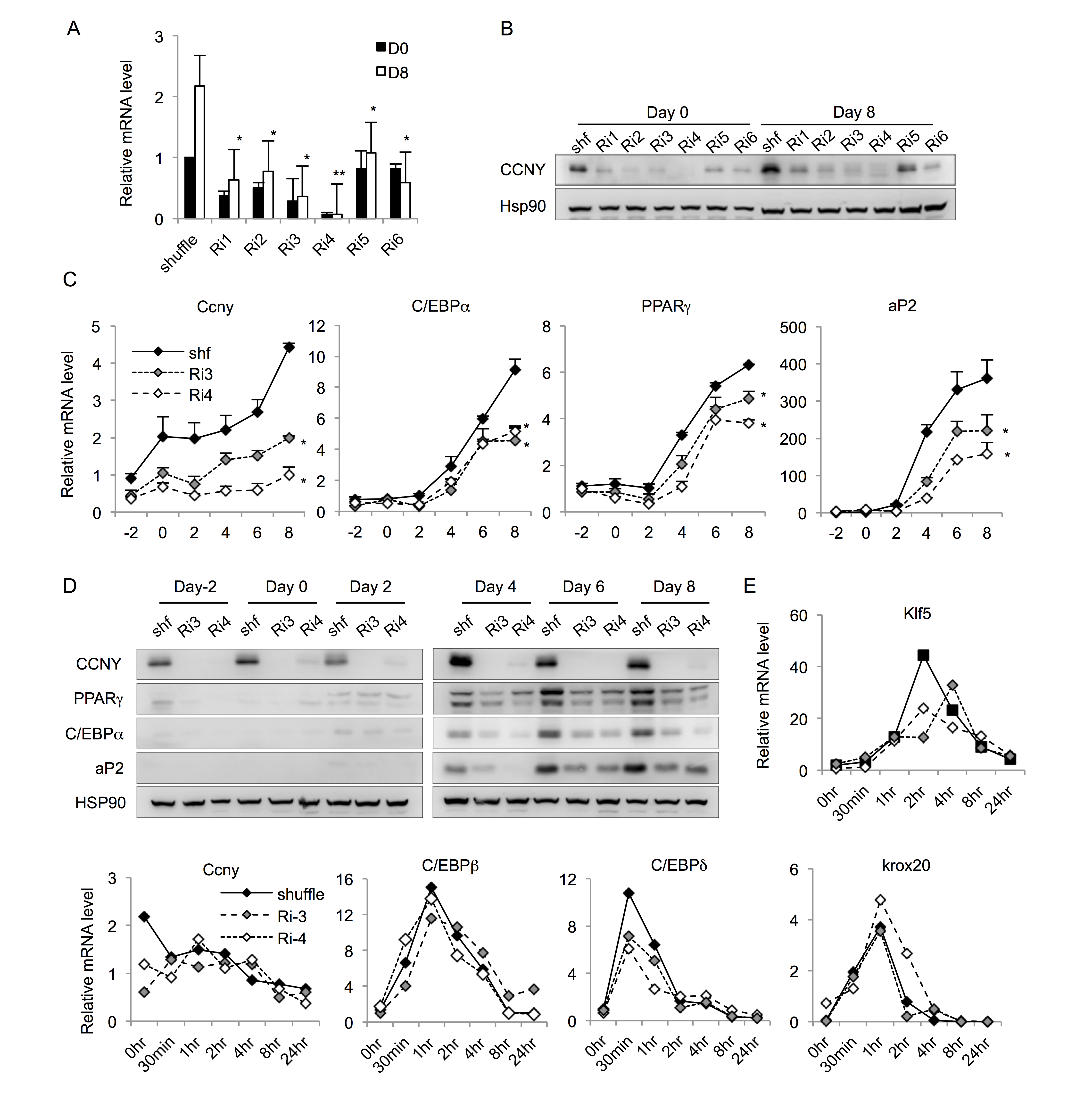

Supplement: S4 Fig — (TIF) [file pone.0132721.s004.tif]

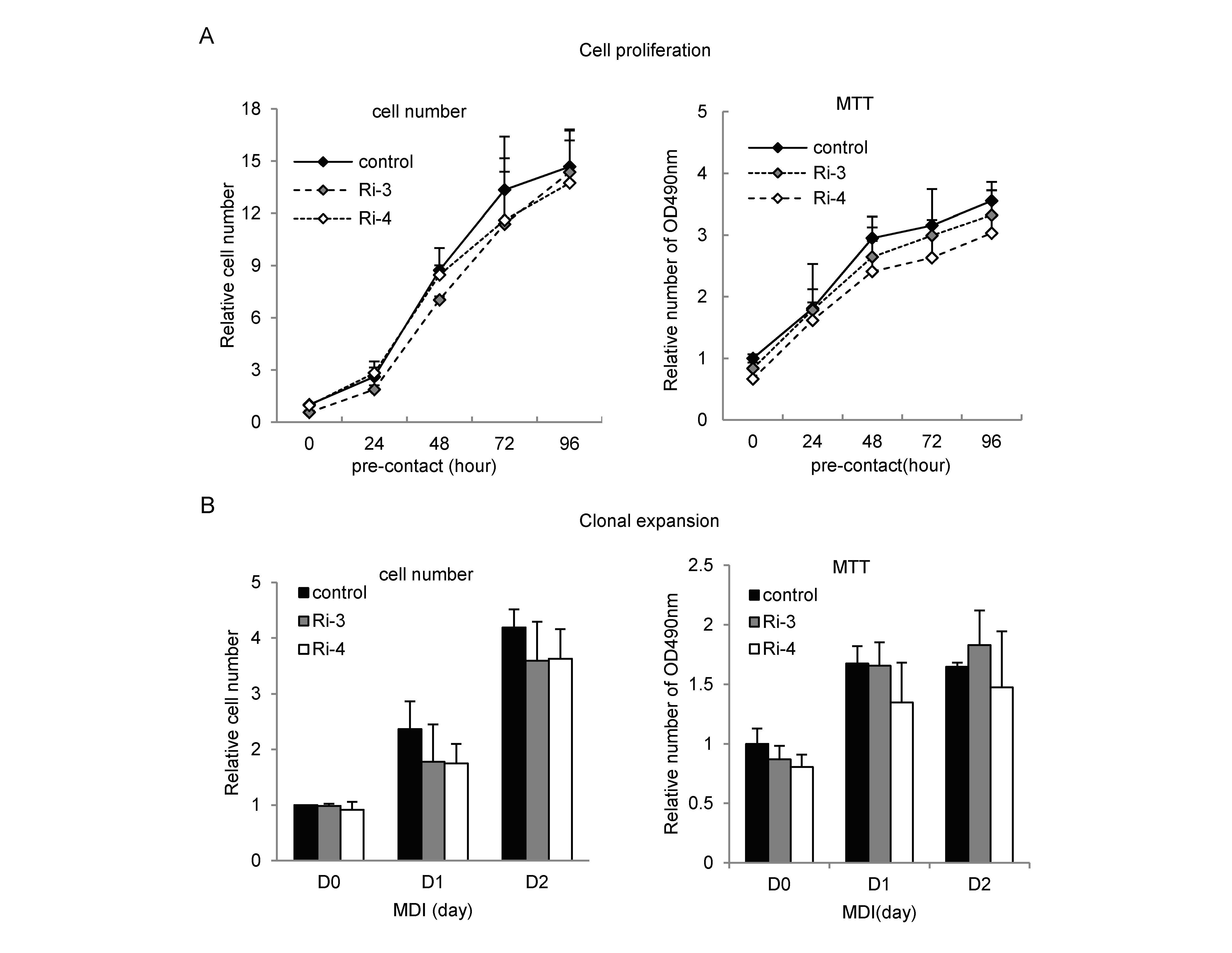

Supplement: S5 Fig — (TIF) [file pone.0132721.s005.tif]
