# Supplementary material for: Cyclin Y Is Involved in the Regulation of Adipogenesis and Lipid Production
Source: PLoS One. 2015 Jul 10;10(7):e0132721. doi: 10.1371/journal.pone.0132721 (PMC4498623; doi:10.1371/journal.pone.0132721)
Supplement: S1 Table — (DOCX) [file pone.0132721.s006.docx]

**S1 Table. The primer pairs used in the text.**

| Gene | Forward primer (5' - 3') | Reverse primer (5' - 3') |
| --- | --- | --- |
| mC/EBPα | CCCAGCGGTGCCTTGTGC | TCCTTCCCCCAGCCGTTAGTG |
| mC/EBPβ | TGGACACGGGACTGACGCAACACA | TCAACAACCCCGCAGGAACATCTT |
| mC/EBPδ | GCAGCCCCAAAAGCCAGTAA | GCCGTGCAGATCAGGGAAGG |
| mKlf5 | CATGCCAAGTCAGTTTCTTCC | TGTGCAACCATTATAATCGCAG |
| mKrox-20 | CTTTGACCAGATGAACGGAG | GAGAATTTGCCCATGTAAGTG |
| maP2 | GATGCCTTTGTGGGAACCTG | TCCTGTCGTCTGCGGTGATT |
| mPPARγ | TGTCGGTTTCAGAAGTGCCTTG | TTCAGCTGGTCGATATCACTGGAG |
| mGAPDH | TGGCAAAGTGGAGATTGTTGCC | AAGATGGTGATGGGCTTCCCG |
| m*Ccny* | CAGGGACCCAGATGGAAGGAT | TTGGCATACACACTGGAAGG |
| mFasn | CACTGCATTGACGGCCGGGT | GGACAAGCCCAGGCTGCGAG |
| mSCD | GCCAGACCGGGCTGAACACC | TGGTGTAGGCGCGTGGCGGA |
| mLPK | GCAGCAGTATGGAAGGGCCAGC | CCATAGCTGCGGGCAGTTGCT |
| Pgc1α | AAGTGTGGAACTCTCTGGAACTG | GGGTTATCTTGGTTGGCTTTATG |
| Ucp1 | ACTGCCACACCTCCAGTCATT | CTTTGCCTCACTCAGGATTGG |
| Ucp3 | ACTCCAGCGTCGCCATCAGGATTCT | TAAACAGGTGAGACTCCAGCAACTT |
| Adipoq | GGATGCTACTGTTGCAAGCTC | TCCTGTCATTCCAACATCTCC |
| Adipor1 | GCATCTCTGCCATCATTGTG | TGGACACACCATAGAAGTGGAC |
| Adipor2 | GAGCCCAGCTTAGAGACACCT | TAGCACATCGTGAGGGATCA |
| Primer pair a | CTTTTTAATTTAGAAGACACAGGT | TCAATATATCACTTGGTTCCCTTA |
| Primer pair b | TACGTTTATCTGGTGTTTCAT | TCTCGCCAGTGACCCACAC |
| Primer pair c | GAGAAGCGCACTGGTGTCTA | CTTGCAACATTTCCGCCTCT |
| Primer pair d | CTGCAAAGTGGTGTATTGTGGT | TGCCTATCAACTGCACACACTG |
